# Biased Transmission of Sex Chromosomes in the Aphid *Myzus persicae* Is Not Associated with Reproductive Mode

**DOI:** 10.1371/journal.pone.0116348

**Published:** 2014-12-30

**Authors:** Alex C. C. Wilson, Ryan N. Delgado, Christoph Vorburger

**Affiliations:** 1 Department of Ecology and Evolutionary Biology, University of Arizona, Tucson, Arizona, United States of America; 2 Institute of Integrative Biology, ETH Zürich, Zürich, Switzerland; 3 Eawag, Swiss Federal Institute of Aquatic Science and Technology, Dübendorf, Switzerland; Centre National de la Recherche Scientifique & University of Nice Sophia-Antipolis, France

## Abstract

Commonly, a single aphid species exhibits a wide range of reproductive strategies including cyclical parthenogenesis and obligate parthenogenesis. Sex determination in aphids is chromosomal; females have two X chromosomes, while males have one. X chromosome elimination at male production is generally random, resulting in equal representation of both X chromosomes in sons. However, two studies have demonstrated deviations from randomness in some lineages. One hypothesis to account for such deviations is that recessive deleterious mutations accumulate during bouts of asexual reproduction and affect male viability, resulting in overrepresentation of males with the least deleterious of the two maternal X chromosomes. This hypothesis results in a testable prediction: X chromosome transmission bias will increase with time spent in the asexual phase and should therefore be most extreme in the least sexual aphid life cycle class. Here we test this prediction in *Myzus persicae*. We used multiple heterozygous X-linked microsatellite markers to screen 1085 males from 95 lines of known life cycle. We found significant deviations from equal representation of X chromosomes in 15 lines; however, these lines included representatives of all life cycles. Our results are inconsistent with the hypothesis that deviations from randomness are attributable to mutation accumulation.

## Introduction

Unequal, or non-Mendelian, representation of the two X chromosomes in male progeny of a single female is unexpectedly common in aphids [1]–[3]. Several mechanisms have been proposed to explain these patterns, including mutation accumulation [1], [4], genetic imprinting of paternal X chromosomes [1] and meiotic drive [2]. Here we test the mutation accumulation hypothesis by genotyping males at X-linked microsatellite markers from multiple *Myzus persicae* lines that have been characterized for life cycle.

Annual cycles within a single aphid species frequently encompass cyclical parthenogenesis (holocyclic - a single sexual generation producing over-wintering eggs followed by numerous parthenogenetic generations during the growth season), obligate parthenogenesis (anholocyclic), obligate parthenogenesis with male production (androcyclic) and intermediate, a “bet-hedging” strategy that involves investment in both sexual and asexual reproduction over the winter months [5]. Parthenogenetic females produce male aphids by a form of arrhenotoky [6]. Males are XO, having two sets of autosomes and only a single X chromosome, whereas female aphids carry a full diploid chromosome complement [6].

During periods of parthenogenetic reproduction, the mutational load of an asexual lineage will increase through the action of Muller’s ratchet [7]. Female aphids have two X chromosomes. Thus, mutant recessive deleterious alleles on aphid X chromosomes will have little effect on female fitness, but, because males are hemizygous for the X chromosome and X chromosomes comprise about one third of an aphid’s genome ([8], A. C. C. Wilson unpublished data), mutation accumulation may strongly affect male fitness. Under a null model, mutations accumulate randomly and thus the mutational load of the two X chromosomes is likely to differ and this may translate into differential viability of male embryos inheriting either X chromosome. In the extreme case, one X chromosome will not be represented in sons if it carries a recessive lethal mutation. Thus, according to the principles of Muller’s ratchet we expect X chromosome transmission bias to increase during time spent in the asexual phase because of the cumulative probability of mutations that are severely deleterious or lethal to their hemizygous male bearers occurring on one of the X chromosomes. Therefore, under the mutation accumulation hypothesis, non-Mendelian transmission of X chromosomes should be more common in the least sexual aphid life cycle classes (male-producing obligate parthenogens) than in those lines that experience regular bouts of sexual reproduction (cyclical parthenogens), because on average, the former have a longer history of clonal inheritance and lack recombination. After sufficient time in the asexual phase, non-overlapping severely deleterious or lethal mutations may even accumulate on both X chromosomes and the lineage would cease male production entirely, becoming genetically constrained to asexuality [2].

## Materials and Methods

### Source of *M. persicae* males

The Australian *M. persicae* lines used in this study were collected in March and April 2002 in central and eastern Victoria, Australia, in a study that examined variation in reproductive mode and genotype across environmental gradients ([9], and see Table S1 in S1 File). In that study the life cycle of all isofemale lines was characterized experimentally and the males produced during that study are the males we have genotyped here. Collection details of 17 *M. persicae* lines collected in the United States are listed in Table 1. These lines were characterized for life cycle following the method of Vorburger et al. [9]. Continuous parthenogenetic reproduction allows successful aphid clones to become locally or regionally abundant such that the same clone can be collected in multiple random field samples. Two or more individual aphids that share identical multilocus genotypes at several highly variable genetic markers are determined to be members of the same superclone. Here we identified one superclone represented by three isofemale lines in our United States collections and nine superclones represented by between two and ten isofemale lines in our Australian collections.

**Table 1:** Genotype, life cycle and collection details of North American *M. persicae* lines.

Line	Genotype	Life cycle	Locality	Host	Date	Coordinates
D001	*USA001*	Androcyclic	Davis, CA	Broccoli	10/14/03	38.554°, 121.738°
D007.2	*USA029*	Androcyclic	Davis, CA	Cauliflower	10/15/03	38.554°, 121.738°
F003	*USA023*	Androcyclic	Freeville, NY	Squash	8/20/03	42.513°, 76.346°
F006	*USA025*	Androcyclic	Freeville, NY	Potato	9/6/03	42.513°, 76.346°
F008	*USA026*	Androcyclic	Freeville, NY	Potato	9/6/03	42.513°, 76.346°
F011	*USA027*	Androcyclic	Freeville, NY	Potato	9/6/03	42.513°, 76.346°
G007	*USA032*	Androcyclic	Geneva, NY	Pepper	8/19/03	42.879°, 76.993°
T004	*USA004*	Androcyclic	Tucson, AZ	Cabbage	12/03	32.222°, 110.926°
T006	*USA020*	Androcyclic	Tucson, AZ	Cabbage	12/03	32.222°, 110.926°
F002	*USA022*	Intermediate	Freeville, NY	Squash	8/20/03	42.513°, 76.346°
F004	*USA024*	Intermediate	Freeville, NY	Potato	9/6/03	42.513°, 76.346°
T001	*USA003*	Intermediate	Tucson, AZ	Cabbage	12/03	32.222°, 110.926°
T005	*USA003*	Intermediate	Tucson, AZ	Cabbage	12/03	32.222°, 110.926°
F001	*USA021*	Holocyclic	Freeville, NY	Squash	8/20/03	42.513°, 76.346°
F012	*USA028*	Holocyclic	Freeville, NY	Potato	9/6/03	42.513°, 76.346°
G003	*USA030*	Holocyclic	Geneva, NY	Pepper	8/19/03	42.879°, 76.993°
G006	*USA031*	Holocyclic	Geneva, NY	Pepper	8/19/03	42.879°, 76.993°
G009	*USA033*	Holocyclic	Geneva, NY	Pepper	8/19/03	42.879°, 76.993°
G010	*USA034*	Holocyclic	Geneva, NY	Pepper	8/19/03	42.879°, 76.993°

Davis and Tucson lines were collected by ACCW. Freeville lines were collected by Georg Jander and the Geneva lines by Brian Nault. Aphids were either collected from public, unprotected land for which no permit was required (roadside verges), from a University campus with which the authors were associated or from private land (a vegetable garden), for which the owner’s permission to collect was obtained prior to accessing the land.

### Characterization of X chromosomes

Single aphid DNA extractions were performed for one asexual female and 4–29 males per isofemale line using a chelex extraction protocol [10]. Restricting the number of males genotyped per line means that we can only reliably detect strong transmission biases; however, this approach allowed us to analyze a large number of lines for comparison of life cycles, which is the main focus of this study. We designed a single multiplexed PCR reaction that amplified the following four X-linked microsatellite loci: M86 from [10] and loci S17b, myz3 and myz25 from [11]. The forward primers for each locus were fluorescently labeled with the following dyes: NED for S17b and myz25, 6-FAM for M86 and HEX for myz3. PCR reactions were carried out in 10 µl reactions containing 0.5 units of *Taq* DNA polymerase (Eppendorf), 50 mM KCl, 10 mM Tris-HCL pH 8.3, 2.0 mM Mg^2+^, 200 µM of each dNTP, 0.25 µM of primers M86f, M86r, myz3f and myz3r, 62.5 pM of primers S17bf, S17br, myz25f and myz25r and 2 µl of DNA from a 30 µl chelex extraction of a single aphid. PCR reactions were run using 5-dye chemistry on an Applied Biosystems 3730 DNA Analyzer by the Genomic Analysis and Technology Core facility at the University of Arizona. Only loci that were heterozygous in a given isofemale line are informative in distinguishing X chromosomes. For this reason, we initially screened a single female from each isofemale line in the original collection to identify those lines that were heterozygous for at least one of the four X-linked loci. Following this initial screening, males from the 95 lines with at least one heterozygous X-linked locus were genotyped.

### X_1_ and X_2_ terminology

Throughout this paper, we refer to the chromosomes as X_1_ and X_2_. The designation is arbitrary but consistent if multiple lines are members of the same genotype or superclone [5], [9].

### Analysis

For each line, we performed two-tailed *G*-tests to test for deviations from equal representation of both X chromosomes in males. We used *G*-tests rather than more conventional binomial or Chi-square tests because this test statistic allows the simultaneous calculation of a ‘pooled’ and a ‘heterogeneity’ *G* in replicated tests ([12], pp. 715–724). The ability to simultaneous calculate a ‘pooled’ and a ‘heterogeneity’ *G* was valuable for clones represented by multiple isofemale lines, providing a test for an overall bias in X chromosome transmission per clone (pooled) and a test of whether X chromosome transmission was consistent among different isofemale lines belonging to the same clone (heterogeneity). However, the *G*-test becomes inaccurate and anti-conservative for small counts, especially with expected frequencies <5, which applied to about a third of our lines. Therefore, we also provide exact binomial tests and we base our conclusions on transmission biases in individual lines on these. For the pooled and heterogeneity tests we adhered to the *G*-tests as counts were sufficient when pooled over isofemal lines of the same clone.

In order to compare deviations from equal representation of the two X chromosomes among groups of lines belonging to different life cycle categories, we used a generalized linear model with the logit link function and binomial errors on the proportion of the overrepresented X (or any X if numbers were exactly equal). This analysis accounts for the unequal number of males among lines. In addition to the comparison among all three life cycle categories, we also compared ‘sexual’ (cyclically parthenogenetic) vs. ‘asexual’ (male-producing obligately parthenogenetic and intermediate) lines, because we believe that the main divide is between lineages that switch completely to sexual reproduction once per year (parthenogenetic lines that are never older than one growth season) and lineages that are able to reproduce parthenogenetically throughout the winter (parthenogenetic lines that are potentially immortal). Among the latter, male-producing obligate parthenogens and intermediates, the variation in the residual investment into sexual reproduction appears gradual, without any sharp divide between lines that still produced a few sexual females as well as males (intermediates) and those that only produce males (male-producing obligate parthenogens) [9]. Nevertheless, intermediate lines may on average have a shorter history of clonal inheritance than male-producing obligate parthenogens. Thus, we also ran a model comparing just cyclically parthenogenetic and male-producing obligately parthenogenetic lines to compare the two most divergent groups.

Statistical analyses were performed using R version 3.0.2 [13].

## Results

### Deviations from equal representation of X-chromosomes found in all life cycle classes

Of the 95 isofemale lines genotyped, 15 showed significant deviations from equal representation of X_1_ and X_2_ males at *α* = 0.05 (18 when *G*-test is used; Table S2 in S1 File). These include 10/47 (21%) male-producing obligately parthenogenetic lines, 2/15 (13%) intermediate lines and 3/33 (9%) cyclically parthenogenetic lines. This number of significant transmission biases is substantially higher than the approximately five significant results we would expect by chance under *α* = 0.05, and two results remain significant after Bonferroni correction (four when *G*-test is used), even though the relatively small number of males analyzed per line precludes very low *P*-values even when biases are strong.

**Figure 1:**
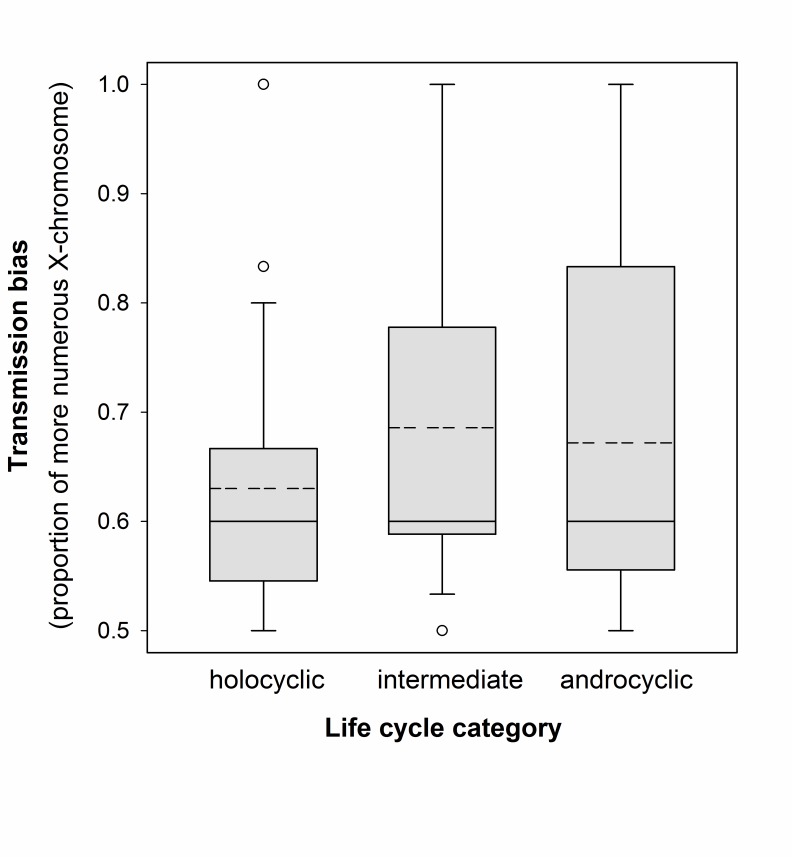
Box plot of X-chromosome transmission bias by life cycle class. Boxes range from the 25^th^ to the 75^th^ percentiles, solid lines within boxes represent medians, dashed lines represent means. Whiskers indicate the 10^th^ and 90^th^ percentiles, circles mark values outside of this range.

Bias in X chromosome transmission expressed as the proportion of males with the more numerous X chromosome did not differ significantly between the three life cycle classes (GLM, χ^2^ = 1.224, df = 2, *P* = 0.542; Fig. 1). Additionally, this result did not change if we eliminated the 15 intermediate lines from the test (χ^2^ = 1.109, df = 1, *P* = 0.296), or if we compared the ‘sexual’ (cyclically parthenogenetic) vs. all ‘asexual’ (male-producing obligately parthenogenetic and intermediate) lines (χ^2^ = 1.224, df = 1, *P* = 0.269).

The mutation accumulation hypothesis states that X-chromosome transmission bias occurs through mortality, thus we expect lines with a strong bias to produce fewer males. However, in the Australian lines for which this information was available, no significant correlation among the total number of males produced and the bias in X transmission was detected (Spearman’s Rho = 0.011, *P* = 0.934).

### Inconsistent patterns of X chromosome elimination within common clones

Thirty-five of the 95 isofemale lines in this study belong to one of nine abundant multilocus asexual genotypes or superclones (Table S3 in S1 File). We also had two holocyclic lines with identical multilocus genotypes (Table S3 in S1 File). These two holocyclic lines had been collected at the very same site and are most likely the product of clonal expansion locally over a single field season and are therefore not considered further below.

For five of the superclones we found patterns of X chromosome inheritance in males to be consistent across lines. For example in the two lines belonging to genotype *21* only X_1_ males were found; neither line produced any X_2_ males. Likewise, all representatives of superclones *25*, *49*, *60*, *61* showed equal representation of both X chromosomes in the samples of males genotyped. However, in four of the nine superclones in this study we found lines with and without significant overrepresentation of one X-chromosome. In both of the two most common clones (multilocus genotypes 45 and 58, each represented by 10 isofemale lines), we even found a mixture of lines without transmission bias, with a significant overrepresentation of X1 and with a significant overrepresentation of X2. In these two cases, the variation in transmission bias is supported by highly significant heterogeneity *G*-tests (multilocus genotype 45: *G* = 32.750, *P*<0.001; multilocus genotype 58: *G* = 27.534, *P* = 0.001). In other words, patterns of over-representation of one X chromosome over the other are sometimes inconsistent within mutationally related lineages.

## Discussion

Non-random transmission of X chromosomes at male production is common and widespread in aphids. This phenomenon has been demonstrated in five species from the tribe Macrosiphini (*Sitobion* near *fragarie* and *Sitobion miscanthi* [2], *Sitobion avenae* [4], *Acyrthosiphon pisum* and *Myzus persicae* (present study)) and one species from the tribe Aphidini (*Rhopalosiphum padi*, [1]). Several hypotheses have been proposed to explain the phenomenon. The first hypothesis proposed, and the one we test here, is the mutation accumulation hypothesis [1], [4]. Other hypotheses include: genetic imprinting of paternal X chromosomes [1] and meiotic drive [2].

### Rejection of the mutation accumulation hypothesis

Using multiple *M. persicae* lines from two continents, we have tested the mutation accumulation hypothesis for the non-random elimination of X chromosomes at male production in aphids. We found strong and significant deviations from equal representation of X chromosomes in *M. persicae* males belonging to all three male-producing aphid life cycle classes and no difference between the strength or frequency of deviations from equal representation in sexual versus asexual lines. Our results therefore do not support the hypothesis that deviations from randomness are the result of mutation accumulation during extended periods of asexual reproduction.

### The segregation distorter hypothesis

Segregation distortion simply refers to any genetic process that results in the non-Mendelian distribution of parental alleles in progeny. Since most common study systems are sexual (e.g. *Drosophila* and the mouse), and segregation distortion in such systems results from processes operating during meiosis, the term “segregation distortion” has been used almost interchangeably with the term “meiotic drive” [14]. Parthenogenetic reproduction in aphids occurs by a modified mitosis [15], and thus application of the term “meiotic drive” in an aphid system would be confusing. However, it is worth noting that segregation distortion in aphids is the mitotic equivalent of meiotic drive.

As we noted in the introduction, a number of hypotheses have been proposed to explain the non-Mendelian inheritance of X chromosomes at male production in aphids, and several of these have now been addressed experimentally. Frantz *et al.* [1] discussed three hypotheses including the idea that nuclear-cytoplasmic incompatibility could be responsible for biases. However, the nuclear-cytoplasmic incompatibility hypothesis was not supported in a study of intraclonal-crosses in *Sitobion* aphids [2]. Therefore, in view of our evidence against the mutation accumulation hypothesis (this study) we are now left with two alternative explanations for the phenomenon of strong biases in the transmission of X chromosomes at male production in aphids: (i) genetic imprinting and (ii) segregation distortion.

The genetic imprinting hypothesis was proposed by Frantz *et al.* [1] and is based on the observation that the nature of aphid parthenogenesis and male production creates an almost unique opportunity for the evolution of a male specific X chromosome. This is because male meiosis is achiasmate [10], aphid parthenogenesis is apomictic (occurring in the absence of genetic recombination, [5]) and males are produced parthenogenetically. This combination of factors means that male-specific X-chromosomes pass unrecombined through males, possibly for long time periods. Therefore, for as long as these X chromosomes are able to avoid the negative effects of mutation accumulation and stochastic processes, they will accumulate genes enhancing male function or, in fact, their own transmission [16]. Whether this opportunity did indeed lead to the imprinting of aphid X chromosomes such that the paternally derived X is preferentially transmitted to male offspring remains to be established. We analyzed field-collected lines and are therefore unable to assign maternal and paternal origin to the two X-chromosomes, but the inconsistent direction of transmission biases among different isofemale lines belonging to the same genetic clone does not support paternal imprinting as the cause of non-random inheritance.

Another possibility of genetic imprinting in aphids has previously been addressed in *M. persicae* by Hales *et al.* [17]. That study was motivated by the fact that aphid spermatogenesis is anomalous in that two kinds of secondary spermatocytes are formed; one possessing an X chromosome, the other lacking one. The spermatocyte with the X chromosome receives more cytoplasm and undergoes a second meiotic division where the spermatocytes without an X degenerate [15]. Autosomes could enhance their transmission by associating with the X chromosome, yet Hales *et al.* [17] found that spermatogenesis in aphids conforms to Mendelian principles.

### Tests of the segregation distorter hypothesis

There are many examples of segregation distorters in Diptera and yet few outside this group [18], [19]. The genetic outcome of a suite of selfish genetic elements, including male-killing agents such as the bacterium *Wolbachia pipientis*, have been mistakenly attributed to the action of segregation distorters (e.g. in the butterflies Danaus chrysippus and Acraea encedon, [18]). In order to demonstrate that the phenomenon we report here, that of non-random elimination of X chromosomes at male production in aphids, results from the action of a segregation distorter it will be necessary to demonstrate the following: (1) that the bias in X chromosome transmission is primary, operating at the formation of the parthenogenetic egg and thus does not involve embryo mortality, and (2) it would be necessary to perform three generations of crossing to characterize the inheritance of the segregation distorter.

Two earlier studies have explicitly examined the point at which the presence of one X chromosome dominates another. The first involved the genotyping of male embryos and juvenile morphs from a lineage of *Sitobion miscanthi* that produced only one type of male [4]. All 47 juvenile males and all 28 male embryos examined in that study were found to carry the same X chromosome indicating that the phenomenon underlying the non-random elimination of the X chromosome in that lineage is acting very early in development. The result in *S. miscanthi* contrasts with the pattern reported for a lineage of *R. padi* where both X chromosomes were represented in embryonic and early larval stage males but only one X chromosome in adult males [1]. Further work is required in lines that show strong deviations from equal representation of X chromosomes in males to determine whether biases in X chromosome transmission are primary. If they are this would provide support for the presence of a segregation distorter in aphids.

### Mitotic recombination?

We have identified a previously unobserved pattern associated with the elimination of X chromosomes at male production in aphid lines – inconsistencies in the transmission bias among multiple isofemale lines belonging to the same multilocus microsatellite genotype. Extremely widespread and abundant genotypes that are derived by apomictic descent from a single foundress are commonly found when highly resolving genetic markers are applied in population studies of aphids [reviewed in 5]. Such genotypes have been referred to as “superclones” [9], or “common genotypes” [5]. The nine superclones examined in this study had been defined in earlier work on the basis of multi-locus microsatellite genotyping over 7–9 loci that included both autosomal and X-linked loci ([9], A. C. C. Wilson, unpublished data). Among those superclones, we identified four examples where the pattern of X transmission was not the same across the different isofemale lines. In some cases, some lines showed significant deviations from equal representation where other lines showed no deviations, and, in the most extreme cases, some superclones had lineages showing no elimination bias as well as significant over-representation of the X_1_ chromosome and significant over-representation of the X_2_ chromosome. These unexpected patterns allow us to conclude several things: (1) superclonal lineages possess cryptic levels of genetic variation, (2) that this cryptic variation, because of its association with a non-Mendelian process is likely to significantly influence the population biology of *M. persicae* and (3) that the factor/gene(s) responsible for the non-random segregation of X chromosomes may move from one X chromosome to the other, during asexual reproduction, possibly via the same (or similar) mechanism that rDNA becomes concentrated on a single X chromosome in long-term asexual aphid lineages [20], [21].

The result that superclonal lineages possess cryptic genetic variation is certainly not surprising; however, the fact that this variation appears to be associated with genetic recombination is unexpected. Aphid parthenogenesis is apomictic; barring mutation and, in the case of males, the elimination of one X chromosome, parthenogenetically produced aphids are genetically identical to their mothers [5]. Considerable efforts have focused on finding evidence of cryptic X chromosome recombination during parthenogenesis in aphids of the tribe Macrosiphini [22], and no recombinants were found in the 1493 individuals screened at multiple X-linked loci. However, mitotic recombination has been implicated in the concentration of rDNA arrays on the ends of only one X chromosome in obligately parthenogenetic aphid lineages [20], [23]. Whilst future experimental work is necessary to determine whether the patterns we report here are attributable to mitotic recombination within superclonal lineages, our observations are consistent with this scenario.

### Summary

Currently, the mechanism of non-random elimination of X chromosomes at male production remains elusive. However, two things are clear; the phenomenon is widespread in aphids and, based on our findings in *M. persicae*, does not appear to result from mutation accumulation during extended periods of asexual reproduction.

## Supporting Information

S1 File **Tables S1–S3. Table S1.** Genotype, life cycle and collection details of Australian *Myzus persicae* lines from (Vorburger *et al*., 2003). Aphids were either collected from public, unprotected land for which no permit was required (roadside verges) or from private land (farms, vegetable gardens), for which the owner’s permission to collect was obtained prior to accessing the land. **Table S2.** The 95 isofemale lines of *Myzus persicae* of which males were genotyped at X-linked microsatellite loci. Counts for both X chromosomes (X_1_, X_2_) are provided together with G-tests as well as exact binomial tests for deviations from equal representation of both chromosomes in the male progeny (without and with strict Bonferroni correction). **Table S3.** Comparisons of X-chromosome transmission to males among independently collected isofemale lines of *Myzus persicae* belonging to the same ‘superclones’ (as identified by identical multilocus microsatellite genotypes). The G-tests for individual lines are identical to those reported in Table S2 in S1 File. The pooled G-tests (‘both/all’) test for deviations from random transmission in each clone as a whole, pooled across male progenies from all isofemale lines, the heterogeneity G-tests indicate whether X chromosome transmission was consistent among different isofemale lines belonging to the same clone.
(DOCX)
