# Correction: Biased Transmission of Sex Chromosomes in the Aphid *Myzus persicae* Is Not Associated with Reproductive Mode

**DOI:** 10.1371/journal.pone.0118524

**Published:** 2015-03-18

**Authors:** The PLOS ONE Staff

## Notice of Republication

This article was republished on January 20, 2015, to replace an incorrect figure. The publisher apologizes for the error. Please download this article again to view the corrected version.
