# Supplementary material for: Biased Transmission of Sex Chromosomes in the Aphid Myzus persicae Is Not Associated with Reproductive Mode
Source: PLoS One. 2014 Dec 30;9(12):e116348. doi: 10.1371/journal.pone.0116348 (PMC4280197; doi:10.1371/journal.pone.0116348)
Supplement: S1 File — Tables S1–S3. Table S1. Genotype, life cycle and collection details of Australian Myzus persicae lines from (Vorburger et al., 2003). Aphids were either collected from public, unprotected land for which no permit was required (roadside verges) or from private land (farms, vegetable gardens), for which the owner’s permission to collect was obtained prior to accessing the land. Table S2. The 95 isofemale lines of Myzus persicae of which males were genotyped at X-linked microsatellite loci. Counts for both X chromosomes (X1, X2) are provided together with G-tests as well as exact binomial tests for deviations from equal representation of both chromosomes in the male progeny (without and with strict Bonferroni correction). Table S3. Comparisons of X-chromosome transmission to males among independently collected isofemale lines of Myzus persicae belonging to the same ‘superclones’ (as identified by identical multilocus microsatellite genotypes). The G-tests for individual lines are identical to those reported in Table S2 in S1 File. The pooled G-tests (‘both/all’) test for deviations from random transmission in each clone as a whole, pooled across male progenies from all isofemale lines, the heterogeneity G-tests indicate whether X chromosome transmission was consistent among different isofemale lines belonging to the same clone. (DOCX) [file pone.0116348.s001.docx]

**Supporting Information**

**Table S1.** Genotype, life cycle and collection details of Australian *Myzus persicae* lines from (Vorburger *et al*., 2003). Aphids were either collected from public, unprotected land for which no permit was required (roadside verges) or from private land (farms, vegetable gardens), for which the owner’s permission to collect was obtained prior to accessing the land.

| Line | Genotype | Life cycle | Locality | Host | Date | Coordinates |
| --- | --- | --- | --- | --- | --- | --- |
| C03 | *58* | androcyclic | Werribee, VIC | *Capsicum annuum* | 3/7/02 | -37.903°, 144.658° |
| C11 | *50* | androcyclic | Pheasant Creek, VIC | *Capsicum annuum* | 3/12/02 | -37.482°, 145.289° |
| C14 | *45* | androcyclic | Pheasant Creek, VIC | *Capsicum annuum* | 3/12/02 | -37.482°, 145.289° |
| C26 | *53* | androcyclic | Silvan, VIC | *Capsicum annuum* | 3/14/02 | -37.824°, 145.421° |
| C63 | *58* | androcyclic | Shepparton, VIC | *Capsicum annuum* | 4/1/02 | -36.325°, 145.351° |
| Cap01 | *16* | androcyclic | Kinglake, VIC | *Capsicum annuum* | 3/12/02 | -37.533°, 145.341° |
| Cap03 | *45* | androcyclic | Kinglake, VIC | *Capsicum annuum* | 3/12/02 | -37.533°, 145.341° |
| Cap21 | *45* | androcyclic | Narbethong, VIC | *Capsicum annuum* | 3/18/02 | -37.564°, 145.657° |
| Cap31 | *43* | androcyclic | Myrtleford, VIC | *Capsicum annuum* | 4/2/02 | -36.562°, 146.726° |
| M03 | *58* | androcyclic | Werribee, VIC | *Malva sp.* | 3/7/02 | -37.903°, 144.658° |
| M08 | *57* | androcyclic | Werribee, VIC | *Malva sp.* | 3/7/02 | -37.903°, 144.658° |
| M22 | *45* | androcyclic | Monbulk, VIC | *Malva sp.* | 3/14/02 | -37.874°, 145.407° |
| M50 | *60* | androcyclic | Bacchus Marsh, VIC | *Malva sp.* | 3/25/02 | -37.675°, 144.439° |
| M56 | *61* | androcyclic | Bacchus Marsh, VIC | *Malva sp.* | 3/25/02 | -37.675°, 144.439° |
| M68 | *13* | androcyclic | Shepparton, VIC | *Malva sp.* | 4/1/02 | -36.325°, 145.351° |
| M70 | *21* | androcyclic | Bundoora, VIC | *Malva sp.* | 5/7/02 | -37.697°, 145.059° |
| Rad10 | *8* | androcyclic | Silvan, VIC | *Raphanus raphanistrum* | 3/14/02 | -37.824°, 145.421° |
| Rad12 | *49* | androcyclic | Silvan, VIC | *Raphanus raphanistrum* | 3/14/02 | -37.824°, 145.421° |
| Rad17 | *54* | androcyclic | Monbulk, VIC | *Raphanus raphanistrum* | 3/14/02 | -37.874°, 145.407° |
| Rad19 | *48* | androcyclic | Monbulk, VIC | *Raphanus raphanistrum* | 3/14/02 | -37.874°, 145.407° |
| Rad52 | *58* | androcyclic | Ballarat, VIC | *Raphanus raphanistrum* | 3/25/02 | -37.561°, 143.867° |
| Rad53 | *45* | androcyclic | Ballarat, VIC | *Raphanus raphanistrum* | 3/25/02 | -37.561°, 143.867° |
| Rad60 | *43* | androcyclic | Ballarat, VIC | *Raphanus raphanistrum* | 3/25/02 | -37.561°, 143.867° |
| Rad79 | *3* | androcyclic | Brigth, VIC | *Raphanus raphanistrum* | 4/2/02 | -36.730°, 146.961° |
| Rad80 | *25* | androcyclic | Brigth, VIC | *Raphanus raphanistrum* | 4/2/02 | -36.730°, 146.961° |
| Sp01 | *45* | androcyclic | Narbethong, VIC | *Spinacia oleracea* | 3/18/02 | -37.564°, 145.657° |
| X12 | *49* | androcyclic | Monbulk, VIC | *Solanum nigrum* | 3/14/02 | -37.874°, 145.407° |
| X40 | *60* | androcyclic | Bacchus Marsh, VIC | *Solanum nigrum* | 3/25/02 | -37.675°, 144.439° |
| X43 | *58* | androcyclic | Bacchus Marsh, VIC | *Solanum nigrum* | 3/25/02 | -37.675°, 144.439° |
| X71 | *21* | androcyclic | Bundoora, VIC | *Solanum nigrum* | 5/4/02 | -37.697°, 145.059° |
| Y40 | *61* | androcyclic | Bacchus Marsh, VIC | *Solanum physalifolium* | 3/25/02 | -37.675°, 144.439° |
| Y41 | *58* | androcyclic | Bacchus Marsh, VIC | *Solanum physalifolium* | 3/25/02 | -37.675°, 144.439° |
| Y42 | *58* | androcyclic | Bacchus Marsh, VIC | *Solanum physalifolium* | 3/25/02 | -37.675°, 144.439° |
| Y44 | *58* | androcyclic | Bacchus Marsh, VIC | *Solanum physalifolium* | 3/25/02 | -37.675°, 144.439° |
| Y64 | *25* | androcyclic | Brigth, VIC | *Solanum physalifolium* | 4/2/02 | -36.730°, 146.961° |
| Z02 | *45* | androcyclic | Swift Creek, VIC | *Hirschfeldia incana* | 4/2/02 | -37.124°, 147.695° |
| Z03 | *58* | androcyclic | Ensay, VIC | *Hirschfeldia incana* | 4/2/02 | -37.369°, 147.856° |
| Bp01 | *37* | holocyclic | Monbulk, VIC | *Capsella bursa-pastoris* | 3/14/02 | -37.874°, 145.407° |
| Bp02 | *12* | holocyclic | Monbulk, VIC | *Capsella bursa-pastoris* | 3/14/02 | -37.874°, 145.407° |
| C60 | *52* | holocyclic | Shepparton, VIC | *Capsicum annuum* | 4/1/02 | -36.325°, 145.351° |
| C61 | *40* | holocyclic | Shepparton, VIC | *Capsicum annuum* | 4/1/02 | -36.325°, 145.351° |
| C62 | *40* | holocyclic | Shepparton, VIC | *Capsicum annuum* | 4/1/02 | -36.325°, 145.351° |
| C70 | *36* | holocyclic | Myrtleford, VIC | *Capsicum annuum* | 4/2/02 | -36.562°, 146.726° |
| Cap30 | *67* | holocyclic | Myrtleford, VIC | *Capsicum annuum* | 4/2/02 | -36.562°, 146.726° |
| M53 | *64* | holocyclic | Bacchus Marsh, VIC | *Malva sp.* | 3/25/02 | -37.675°, 144.439° |
| M62 | *71* | holocyclic | Shepparton, VIC | *Malva sp.* | 4/1/02 | -36.325°, 145.351° |
| M64 | *69* | holocyclic | Shepparton, VIC | *Malva sp.* | 4/1/02 | -36.325°, 145.351° |
| M65 | *62* | holocyclic | Shepparton, VIC | *Malva sp.* | 4/1/02 | -36.325°, 145.351° |
| M66 | *17* | holocyclic | Shepparton, VIC | *Malva sp.* | 4/1/02 | -36.325°, 145.351° |
| Rad72 | *68* | holocyclic | Myrtleford, VIC | *Raphanus raphanistrum* | 4/2/02 | -36.562°, 146.726° |
| Rad75 | *10* | holocyclic | Myrtleford, VIC | *Raphanus raphanistrum* | 4/2/02 | -36.562°, 146.726° |
| Rad77 | *66* | holocyclic | Brigth, VIC | *Raphanus raphanistrum* | 4/2/02 | -36.730°, 146.961° |
| X13 | *70* | holocyclic | Monbulk, VIC | *Solanum nigrum* | 3/14/02 | -37.874°, 145.407° |
| X50 | *33* | holocyclic | Shepparton, VIC | *Solanum nigrum* | 4/1/02 | -36.325°, 145.351° |
| X53 | *63* | holocyclic | Shepparton, VIC | *Solanum nigrum* | 4/1/02 | -36.325°, 145.351° |
| X56 | *6* | holocyclic | Shepparton, VIC | *Solanum nigrum* | 4/1/02 | -36.325°, 145.351° |
| Y03 | *7* | holocyclic | Silvan, VIC | *Solanum physalifolium* | 3/14/02 | -37.824°, 145.421° |
| Y52 | *1* | holocyclic | Myrtleford, VIC | *Solanum physalifolium* | 4/2/02 | -36.562°, 146.726° |
| Y54 | *34* | holocyclic | Myrtleford, VIC | *Solanum physalifolium* | 4/2/02 | -36.562°, 146.726° |
| Y55 | *38* | holocyclic | Myrtleford, VIC | *Solanum physalifolium* | 4/2/02 | -36.562°, 146.726° |
| Y56 | *35* | holocyclic | Myrtleford, VIC | *Solanum physalifolium* | 4/2/02 | -36.562°, 146.726° |
| Y57 | *31* | holocyclic | Myrtleford, VIC | *Solanum physalifolium* | 4/2/02 | -36.562°, 146.726° |
| Y61 | *29* | holocyclic | Myrtleford, VIC | *Solanum physalifolium* | 4/2/02 | -36.562°, 146.726° |
| Y62 | *39* | holocyclic | Brigth, VIC | *Solanum physalifolium* | 4/2/02 | -36.730°, 146.961° |
| Bro01 | *45* | intermediate | Pheasant Creek, VIC | *Brassica oleracea* | 3/12/02 | -37.482°, 145.289° |
| C04 | *23* | intermediate | Werribee, VIC | *Capsicum annuum* | 3/7/02 | -37.903°, 144.658° |
| C10 | *4* | intermediate | Pheasant Creek, VIC | *Capsicum annuum* | 3/12/02 | -37.482°, 145.289° |
| M25 | *51* | intermediate | Monbulk, VIC | *Malva sp.* | 3/14/02 | -37.874°, 145.407° |
| M57 | *44* | intermediate | Bacchus Marsh, VIC | *Malva sp.* | 3/25/02 | -37.675°, 144.439° |
| P06 | *45* | intermediate | Kinglake, VIC | *Solanum tuberosum* | 3/12/02 | -37.533°, 145.341° |
| P07 | *73* | intermediate | Kinglake, VIC | *Solanum tuberosum* | 3/12/02 | -37.533°, 145.341° |
| Rad18 | *9* | intermediate | Monbulk, VIC | *Raphanus raphanistrum* | 3/14/02 | -37.874°, 145.407° |
| X11 | *45* | intermediate | Monbulk, VIC | *Solanum nigrum* | 3/14/02 | -37.874°, 145.407° |
| X42 | *15* | intermediate | Bacchus Marsh, VIC | *Solanum nigrum* | 3/25/02 | -37.675°, 144.439° |
| Y01 | *58* | intermediate | Werribee, VIC | *Solanum physalifolium* | 3/7/02 | -37.903°, 144.658° |

**Table S2.** The 95 isofemale lines of *Myzus persicae* of which males were genotyped at X-linked microsatellite loci. Counts for both X chromosomes (X_1_, X_2_) are provided together with G-tests as well as exact binomial tests for deviations from equal representation of both chromosomes in the male progeny (without and with strict Bonferroni correction).

| **Line** | **Life cycle** | **Genotype** | **X_1_ count** | **X_2_ count** | **Total  males** | ***G***  **statistic** | ***P***  **(*G*-test)** | **Sig.**  **(α = 0.05)** | **Sig.**  **(Bonferroni)** | ***P***  **(binomial)** | **Sig.**  **(α = 0.05)** | **Sig.**  **(Bonferroni)** |
| --- | --- | --- | --- | --- | --- | --- | --- | --- | --- | --- | --- | --- |
| D001 | androcyclic | *USA001* | 3 | 7 | 10 | 1.6457 | 0.1996 |  |  | 0.3438 |  |  |
| D007.2 | androcyclic | *USA029* | 12 | 7 | 19 | 1.3314 | 0.2486 |  |  | 0.3593 |  |  |
| F003 | androcyclic | *USA023* | 12 | 2 | 14 | 7.9249 | 0.0049 | * |  | 0.0129 | * |  |
| F006 | androcyclic | *USA025* | 6 | 11 | 17 | 1.4926 | 0.2218 |  |  | 0.3323 |  |  |
| F008 | androcyclic | *USA026* | 6 | 0 | 6 | 8.3178 | 0.0039 | * |  | 0.0313 | * |  |
| F011 | androcyclic | *USA027* | 5 | 4 | 9 | 0.1113 | 0.7386 |  |  | 1.0000 |  |  |
| G007 | androcyclic | *USA032* | 6 | 3 | 9 | 1.0194 | 0.3127 |  |  | 0.5078 |  |  |
| T003 | androcyclic | *USA003* | 5 | 5 | 10 | 0.0000 | 1.0000 |  |  | 1.0000 |  |  |
| T004 | androcyclic | *USA004* | 6 | 4 | 10 | 0.4027 | 0.5257 |  |  | 0.7539 |  |  |
| T006 | androcyclic | *USA020* | 5 | 5 | 10 | 0.0000 | 1.0000 |  |  | 1.0000 |  |  |
| Rad79 | androcyclic | *3* | 4 | 7 | 11 | 0.8286 | 0.3627 |  |  | 0.5488 |  |  |
| Rad10 | androcyclic | *8* | 5 | 4 | 9 | 0.1113 | 0.7386 |  |  | 1.0000 |  |  |
| M68 | androcyclic | *13* | 10 | 15 | 25 | 1.0068 | 0.3157 |  |  | 0.4244 |  |  |
| Cap01 | androcyclic | *16* | 8 | 8 | 16 | 0.0000 | 1.0000 |  |  | 1.0000 |  |  |
| M70 | androcyclic | *21* | 0 | 7 | 7 | 9.7041 | 0.0018 | * |  | 0.0156 | * |  |
| X71 | androcyclic | *21* | 0 | 10 | 10 | 13.8629 | 0.0002 | * | * | 0.0020 | * |  |
| Rad80 | androcyclic | *25* | 7 | 5 | 12 | 0.3349 | 0.5628 |  |  | 0.7744 |  |  |
| Y64 | androcyclic | *25* | 4 | 6 | 10 | 0.4027 | 0.5257 |  |  | 0.7539 |  |  |
| Cap31 | androcyclic | *43* | 8 | 5 | 13 | 0.6986 | 0.4033 |  |  | 0.5811 |  |  |
| Rad60 | androcyclic | *43* | 9 | 1 | 10 | 7.3613 | 0.0067 | * |  | 0.0215 | * |  |
| C14 | androcyclic | *45* | 4 | 3 | 7 | 0.1433 | 0.7050 |  |  | 1.0000 |  |  |
| Cap03 | androcyclic | *45* | 2 | 2 | 4 | 0.0000 | 1.0000 |  |  | 1.0000 |  |  |
| Cap21 | androcyclic | *45* | 2 | 10 | 12 | 5.8221 | 0.0158 | * |  | 0.0386 | * |  |
| M22 | androcyclic | *45* | 1 | 5 | 6 | 2.9110 | 0.0880 |  |  | 0.2188 |  |  |
| Rad53 | androcyclic | *45* | 8 | 1 | 9 | 6.1977 | 0.0128 | * |  | 0.0391 | * |  |
| Sp01 | androcyclic | *45* | 4 | 5 | 9 | 0.1113 | 0.7386 |  |  | 1.0000 |  |  |
| Z02 | androcyclic | *45* | 8 | 10 | 18 | 0.2227 | 0.6370 |  |  | 0.8145 |  |  |
| Rad19 | androcyclic | *48* | 4 | 4 | 8 | 0.0000 | 1.0000 |  |  | 1.0000 |  |  |
| Rad12 | androcyclic | *49* | 4 | 4 | 8 | 0.0000 | 1.0000 |  |  | 1.0000 |  |  |
| X12 | androcyclic | *49* | 4 | 5 | 9 | 0.1113 | 0.7386 |  |  | 1.0000 |  |  |
| C11 | androcyclic | *50* | 5 | 5 | 10 | 0.0000 | 1.0000 |  |  | 1.0000 |  |  |
| C26 | androcyclic | *53* | 6 | 5 | 11 | 0.0910 | 0.7629 |  |  | 1.0000 |  |  |
| Rad17 | androcyclic | *54* | 0 | 17 | 17 | 23.5670 | 0.0000 | * | * | 0.0000 | * | * |
| M08 | androcyclic | *57* | 4 | 5 | 9 | 0.1113 | 0.7386 |  |  | 1.0000 |  |  |
| C03 | androcyclic | *58* | 4 | 8 | 12 | 1.3592 | 0.2437 |  |  | 0.3877 |  |  |
| C63 | androcyclic | *58* | 6 | 0 | 6 | 8.3178 | 0.0039 | * |  | 0.0313 | * |  |
| M03 | androcyclic | *58* | 3 | 6 | 9 | 1.0194 | 0.3127 |  |  | 0.5078 |  |  |
| Rad52 | androcyclic | *58* | 3 | 4 | 7 | 0.1433 | 0.7050 |  |  | 1.0000 |  |  |
| X43 | androcyclic | *58* | 5 | 1 | 6 | 2.9110 | 0.0880 |  |  | 0.2188 |  |  |
| Y41 | androcyclic | *58* | 13 | 16 | 29 | 0.3109 | 0.5771 |  |  | 0.7111 |  |  |
| Y42 | androcyclic | *58* | 0 | 9 | 9 | 12.4767 | 0.0004 | * | * | 0.0039 | * |  |
| Y44 | androcyclic | *58* | 4 | 6 | 10 | 0.4027 | 0.5257 |  |  | 0.7539 |  |  |
| Z03 | androcyclic | *58* | 6 | 3 | 9 | 1.0194 | 0.3127 |  |  | 0.5078 |  |  |
| M50 | androcyclic | *60* | 5 | 4 | 9 | 0.1113 | 0.7386 |  |  | 1.0000 |  |  |
| X40 | androcyclic | *60* | 2 | 7 | 9 | 2.9419 | 0.0863 |  |  | 0.1797 |  |  |
| M56 | androcyclic | *61* | 2 | 3 | 5 | 0.2014 | 0.6536 |  |  | 1.0000 |  |  |
| Y40 | androcyclic | *61* | 6 | 5 | 11 | 0.0910 | 0.7629 |  |  | 1.0000 |  |  |
| F001 | holocyclic | *USA021* | 5 | 4 | 9 | 0.1113 | 0.7386 |  |  | 1.0000 |  |  |
| F012 | holocyclic | *USA028* | 8 | 6 | 14 | 0.2867 | 0.5923 |  |  | 0.7905 |  |  |
| G003 | holocyclic | *USA030* | 3 | 2 | 5 | 0.2014 | 0.6536 |  |  | 1.0000 |  |  |
| G006 | holocyclic | *USA031* | 7 | 4 | 11 | 0.8286 | 0.3627 |  |  | 0.5488 |  |  |
| G009 | holocyclic | *USA033* | 4 | 7 | 11 | 0.8286 | 0.3627 |  |  | 0.5488 |  |  |
| G010 | holocyclic | *USA034* | 9 | 5 | 14 | 1.1589 | 0.2817 |  |  | 0.4240 |  |  |
| Y52 | holocyclic | *1* | 4 | 8 | 12 | 1.3592 | 0.2437 |  |  | 0.3877 |  |  |
| X56 | holocyclic | *6* | 6 | 5 | 11 | 0.0910 | 0.7629 |  |  | 1.0000 |  |  |
| Y03 | holocyclic | *7* | 4 | 4 | 8 | 0.0000 | 1.0000 |  |  | 1.0000 |  |  |
| Rad75 | holocyclic | *10* | 4 | 6 | 10 | 0.4027 | 0.5257 |  |  | 0.7539 |  |  |
| Bp02 | holocyclic | *12* | 10 | 2 | 12 | 5.8221 | 0.0158 | * |  | 0.0386 | * |  |
| M66 | holocyclic | *17* | 8 | 7 | 15 | 0.0667 | 0.7962 |  |  | 1.0000 |  |  |
| Y61 | holocyclic | *29* | 9 | 3 | 12 | 3.1395 | 0.0764 |  |  | 0.1460 |  |  |
| Y57 | holocyclic | *31* | 9 | 5 | 14 | 1.1589 | 0.2817 |  |  | 0.4240 |  |  |
| X50 | holocyclic | *33* | 6 | 4 | 10 | 0.4027 | 0.5257 |  |  | 0.7539 |  |  |
| Y54 | holocyclic | *34* | 9 | 8 | 17 | 0.0589 | 0.8083 |  |  | 1.0000 |  |  |
| Y56 | holocyclic | *35* | 7 | 8 | 15 | 0.0667 | 0.7962 |  |  | 1.0000 |  |  |
| C70 | holocyclic | *36* | 3 | 6 | 9 | 1.0194 | 0.3127 |  |  | 0.5078 |  |  |
| Bp01 | holocyclic | *37* | 5 | 6 | 11 | 0.0910 | 0.7629 |  |  | 1.0000 |  |  |
| Y55 | holocyclic | *38* | 0 | 15 | 15 | 20.7944 | 0.0000 | * | * | 0.0001 | * | * |
| Y62 | holocyclic | *39* | 9 | 9 | 18 | 0.0000 | 1.0000 |  |  | 1.0000 |  |  |
| C61 | holocyclic | *40* | 12 | 4 | 16 | 4.1860 | 0.0408 | * |  | 0.0768 |  |  |
| C62 | holocyclic | *40* | 5 | 4 | 9 | 0.1113 | 0.7386 |  |  | 1.0000 |  |  |
| C60 | holocyclic | *52* | 5 | 9 | 14 | 1.1589 | 0.2817 |  |  | 0.4240 |  |  |
| M65 | holocyclic | *62* | 6 | 9 | 15 | 0.6041 | 0.4370 |  |  | 0.6072 |  |  |
| X53 | holocyclic | *63* | 12 | 6 | 18 | 2.0388 | 0.1533 |  |  | 0.2379 |  |  |
| M53 | holocyclic | *64* | 7 | 7 | 14 | 0.0000 | 1.0000 |  |  | 1.0000 |  |  |
| Rad77 | holocyclic | *66* | 6 | 6 | 12 | 0.0000 | 1.0000 |  |  | 1.0000 |  |  |
| Cap30 | holocyclic | *67* | 8 | 0 | 8 | 11.0904 | 0.0009 | * |  | 0.0078 | * |  |
| Rad72 | holocyclic | *68* | 4 | 6 | 10 | 0.4027 | 0.5257 |  |  | 0.7539 |  |  |
| M64 | holocyclic | *69* | 4 | 6 | 10 | 0.4027 | 0.5257 |  |  | 0.7539 |  |  |
| X13 | holocyclic | *70* | 7 | 3 | 10 | 1.6457 | 0.1996 |  |  | 0.3438 |  |  |
| M62 | holocyclic | *71* | 7 | 10 | 17 | 0.5322 | 0.4657 |  |  | 0.6291 |  |  |
| F002 | intermediate | *USA022* | 6 | 4 | 10 | 0.4027 | 0.5257 |  |  | 0.7539 |  |  |
| F004 | intermediate | *USA024* | 4 | 6 | 10 | 0.4027 | 0.5257 |  |  | 0.7539 |  |  |
| T001 | intermediate | *USA003* | 4 | 6 | 10 | 0.4027 | 0.5257 |  |  | 0.7539 |  |  |
| T005 | intermediate | *USA003* | 14 | 5 | 19 | 4.4389 | 0.0351 | * |  | 0.0636 |  |  |
| C10 | intermediate | *4* | 10 | 13 | 23 | 0.3924 | 0.5310 |  |  | 0.6776 |  |  |
| Rad18 | intermediate | *9* | 4 | 4 | 8 | 0.0000 | 1.0000 |  |  | 1.0000 |  |  |
| X42 | intermediate | *15* | 10 | 8 | 18 | 0.2227 | 0.6370 |  |  | 0.8145 |  |  |
| C04 | intermediate | *23* | 7 | 10 | 17 | 0.5322 | 0.4657 |  |  | 0.6291 |  |  |
| M57 | intermediate | *44* | 13 | 3 | 16 | 6.7382 | 0.0094 | * |  | 0.0213 | * |  |
| Bro01 | intermediate | *45* | 2 | 6 | 8 | 2.0930 | 0.1480 |  |  | 0.2891 |  |  |
| P06 | intermediate | *45* | 6 | 0 | 6 | 8.3178 | 0.0039 | * |  | 0.0313 | * |  |
| X11 | intermediate | *45* | 5 | 0 | 5 | 6.9315 | 0.0085 | * |  | 0.0625 |  |  |
| M25 | intermediate | *51* | 7 | 2 | 9 | 2.9419 | 0.0863 |  |  | 0.1797 |  |  |
| Y01 | intermediate | *58* | 3 | 2 | 5 | 0.2014 | 0.6536 |  |  | 1.0000 |  |  |
| P07 | intermediate | *73* | 6 | 4 | 10 | 0.4027 | 0.5257 |  |  | 0.7539 |  |  |

**Table S3.** Comparisons of X-chromosome transmission to males among independently collected isofemale lines of *Myzus persicae* belonging to the same 'superclones' (as identified by identical multilocus microsatellite genotypes). The G-tests for individual lines are identical to those reported in Table S2. The pooled G-tests ('both/all') test for deviations from random transmission in each clone as a whole, pooled across male progenies from all isofemale lines, the heterogeneity G-tests indicate whether X chromosome transmission was consistent among different isofemale lines belonging to the same clone.

| **Line** | **Life cycle** | **Genotype** | **X1 count** | **X2 count** | **Total males** | ***G*** | ***P*** | **Heterogeneity *G*** | **Heterogeneity *P*** |
| --- | --- | --- | --- | --- | --- | --- | --- | --- | --- |
| M70 | androcyclic | 21 | 0 | 7 | 7 | 9.7041 | 0.0018 |  |  |
| X71 | androcyclic | 21 | 0 | 10 | 10 | 13.8629 | 0.0002 |  |  |
| both |  |  | 0 | 17 | 17 | 23.5670 | 0.0000 | 0.0000 | 0.9989 |
|  |  |  |  |  |  |  |  |  |  |
| Rad80 | androcyclic | 25 | 7 | 5 | 12 | 0.3349 | 0.5628 |  |  |
| Y64 | androcyclic | 25 | 4 | 6 | 10 | 0.4027 | 0.5257 |  |  |
| both |  |  | 11 | 11 | 22 | 0.0000 | 1.0000 | 0.7376 | 0.3904 |
|  |  |  |  |  |  |  |  |  |  |
| C61 | holocyclic | 40 | 12 | 4 | 16 | 4.1860 | 0.0408 |  |  |
| C62 | holocyclic | 40 | 5 | 4 | 9 | 0.1113 | 0.7386 |  |  |
| both |  |  | 17 | 8 | 25 | 3.3139 | 0.0687 | 0.9834 | 0.3214 |
|  |  |  |  |  |  |  |  |  |  |
| Cap31 | androcyclic | 43 | 8 | 5 | 13 | 0.6986 | 0.4033 |  |  |
| Rad60 | androcyclic | 43 | 9 | 1 | 10 | 7.3613 | 0.0067 |  |  |
| both |  |  | 17 | 6 | 23 | 5.4824 | 0.0192 | 2.5775 | 0.1084 |
|  |  |  |  |  |  |  |  |  |  |
| C14 | androcyclic | 45 | 4 | 3 | 7 | 0.1433 | 0.7050 |  |  |
| Cap03 | androcyclic | 45 | 2 | 2 | 4 | 0.0000 | 1.0000 |  |  |
| Cap21 | androcyclic | 45 | 2 | 10 | 12 | 5.8221 | 0.0158 |  |  |
| M22 | androcyclic | 45 | 1 | 5 | 6 | 2.9110 | 0.0880 |  |  |
| Rad53 | androcyclic | 45 | 8 | 1 | 9 | 6.1977 | 0.0128 |  |  |
| Sp01 | androcyclic | 45 | 4 | 5 | 9 | 0.1113 | 0.7386 |  |  |
| Z02 | androcyclic | 45 | 8 | 10 | 18 | 0.2227 | 0.6370 |  |  |
| Bro01 | intermediate | 45 | 2 | 6 | 8 | 2.0930 | 0.1480 |  |  |
| P06 | intermediate | 45 | 6 | 0 | 6 | 8.3178 | 0.0039 |  |  |
| X11 | intermediate | 45 | 5 | 0 | 5 | 6.9315 | 0.0085 |  |  |
| all |  |  | 42 | 42 | 84 | 0.0000 | 1.0000 | 32.7504 | 0.0001 |
|  |  |  |  |  |  |  |  |  |  |
| Rad12 | androcyclic | 49 | 4 | 4 | 8 | 0.0000 | 1.0000 |  |  |
| X12 | androcyclic | 49 | 4 | 5 | 9 | 0.1113 | 0.7386 |  |  |
| both |  |  | 8 | 9 | 17 | 0.0589 | 0.8083 | 0.0525 | 0.8188 |
|  |  |  |  |  |  |  |  |  |  |
| C03 | androcyclic | 58 | 4 | 8 | 12 | 1.3592 | 0.2437 |  |  |
| C63 | androcyclic | 58 | 6 | 0 | 6 | 8.3178 | 0.0039 |  |  |
| M03 | androcyclic | 58 | 3 | 6 | 9 | 1.0194 | 0.3127 |  |  |
| Rad52 | androcyclic | 58 | 3 | 4 | 7 | 0.1433 | 0.7050 |  |  |
| X43 | androcyclic | 58 | 5 | 1 | 6 | 2.9110 | 0.0880 |  |  |
| Y41 | androcyclic | 58 | 13 | 16 | 29 | 0.3109 | 0.5771 |  |  |
| Y42 | androcyclic | 58 | 0 | 9 | 9 | 12.4767 | 0.0004 |  |  |
| Y44 | androcyclic | 58 | 4 | 6 | 10 | 0.4027 | 0.5257 |  |  |
| Z03 | androcyclic | 58 | 6 | 3 | 9 | 1.0194 | 0.3127 |  |  |
| Y01 | intermediate | 58 | 3 | 2 | 5 | 0.2014 | 0.6536 |  |  |
| all |  |  | 47 | 55 | 102 | 0.6281 | 0.4281 | 27.5336 | 0.0011 |
|  |  |  |  |  |  |  |  |  |  |
| M50 | androcyclic | 60 | 5 | 4 | 9 | 0.1113 | 0.7386 |  |  |
| X40 | androcyclic | 60 | 2 | 7 | 9 | 2.9419 | 0.0863 |  |  |
| both |  |  | 7 | 11 | 18 | 0.8964 | 0.3438 | 2.1569 | 0.1419 |
|  |  |  |  |  |  |  |  |  |  |
| M56 | androcyclic | 61 | 2 | 3 | 5 | 0.2014 | 0.6536 |  |  |
| Y40 | androcyclic | 61 | 6 | 5 | 11 | 0.0910 | 0.7629 |  |  |
| both |  |  | 8 | 8 | 16 | 0.0000 | 1.0000 | 0.2924 | 0.5887 |
|  |  |  |  |  |  |  |  |  |  |
| T003 | androcyclic | USA003 | 5 | 5 | 10 | 0.0000 | 1.0000 |  |  |
| T001 | intermediate | USA003 | 4 | 6 | 10 | 0.4027 | 0.5257 |  |  |
| T005 | intermediate | USA003 | 14 | 5 | 19 | 4.4389 | 0.0351 |  |  |
| all |  |  | 23 | 16 | 39 | 1.2632 | 0.2610 | 3.5784 | 0.1671 |
|  |  |  |  |  |  |  |  |  |  |

**References**

Vorburger C, Lancaster M, Sunnucks P (2003) Environmentally-related patterns of reproductive modes in the aphid *Myzus persicae* and the predominance of two 'superclones' in Victoria, Australia. Molecular Ecology 12: 3493-3504.
